# Why Are Seizures Rare in Rapid Eye Movement Sleep? Review of the Frequency of Seizures in Different Sleep Stages

**DOI:** 10.1155/2013/932790

**Published:** 2013-06-18

**Authors:** Marcus Ng, Milena Pavlova

**Affiliations:** ^1^Department of Neurology, Epilepsy Service, Massachusetts General Hospital, 55 Fruit Street, Boston, MA 02114, USA; ^2^Department of Neurology, Division of Epilepsy, EEG, and Sleep Neurology, Brigham and Women's-Faulkner Hospital, 1153 Centre Street, Boston, MA 02130, USA

## Abstract

Since the formal characterization of sleep stages, there have been reports that seizures may preferentially occur in certain phases of sleep. Through ascending cholinergic connections from the brainstem, rapid eye movement (REM) sleep is physiologically characterized by low voltage fast activity on the electroencephalogram, REMs, and muscle atonia. Multiple independent studies confirm that, in REM sleep, there is a strikingly low proportion of seizures (~1% or less). We review a total of 42 distinct conventional and intracranial studies in the literature which comprised a net of 1458 patients. Indexed to duration, we found that REM sleep was the most protective stage of sleep against focal seizures, generalized seizures, focal interictal discharges, and two particular epilepsy syndromes. REM sleep had an additional protective effect compared to wakefulness with an average 7.83 times fewer focal seizures, 3.25 times fewer generalized seizures, and 1.11 times fewer focal interictal discharges. In further studies REM sleep has also demonstrated utility in localizing epileptogenic foci with potential translation into postsurgical seizure freedom. Based on emerging connectivity data in sleep, we hypothesize that the influence of REM sleep on seizures is due to a desynchronized EEG pattern which reflects important connectivity differences unique to this sleep stage.

## 1. Introduction

A bidirectional relationship between epilepsy and sleep has been observed since the time of Hippocrates [1]. It was not until the first formal characterization of sleep stages that this relationship became successively attuned to each specific sleep stage. It became apparent that seizures may preferentially occur during certain phases of sleep with the least likelihood of occurrence in rapid eye movement (REM) sleep. The purpose of this review is to focus on the impact of REM sleep on seizures. We discuss REM sleep physiology, a review of the available literature regarding seizures during REM sleep, and a consideration of the potential mechanisms which may underlie this intriguing but often overlooked phenomenon.

## 2. REM Sleep Physiology

Based on a wealth of animal and human data accumulated since the discovery of REM sleep in 1953 [2], an exciting and coherent model of REM sleep physiology has emerged. In the pontomesencephalic junction of the brainstem, there are two populations of cholinergic neurons in the laterodorsal tegmentum (LDT) and pedunculopontine tegmentum (PPT) [3]. Within these populations, there is a subset of cells that are most active in REM sleep, as well as another subset, which is active in both REM sleep and wakefulness [4–7]. 

Of the neurons whose spontaneous firing rate is highest in REM sleep, some exhibit a spontaneous bursting depolarization pattern due to a “low threshold spike” inward calcium current [8]. Through muscarinic greater than nicotinic acetylcholine receptors [9], connections from the LDT and PPT excite populations of neurons in the pontine reticular formation (PRF) and mesencephalic reticular formation (MRF) which serve as “effector cells” responsible for the following dissociable characteristics of REM sleep:

1. low voltage fast electroencephalographic (EEG) activity,
2. rapid eye movements (REMs),
3. muscle atonia.

Low voltage fast activity on the EEG is due largely to depolarization of the thalamus by cholinergic MRF neurons [8]. Thalamic activation allows transmission of information to the cortex and subsequent EEG desynchronization which contrasts with the generally synchronized high-voltage activity of slow-wave sleep [10]. There is also evidence that another cholinergic subpopulation ventral to the dorsolateral PRF depolarizes the thalamus; however, the resultant EEG resembles more the waking state than REM sleep [11]. Furthermore, the LDT and PPT may directly activate cholinergic centers in the basal forebrain which has further excitatory connections to hippocampus and cortex [12–15]. In addition, there may be an element of cortical disinhibition as the basal forebrain also contains gamma-aminobutyric acid (GABA) ergic neurons which may be stimulated to deactivate inhibitory interneurons with further projections to hippocampus and cortex [16, 17].

Rapid eye movements are heralded by discharges, known as pontogeniculooccipital (PGO) waves, from a dorsorostral subpopulation of cholinergic PRF neurons which project to the occipital lobe via the lateral geniculate nucleus (LGN). The presence of PGO waves precedes REMs by 3–5 waves and low voltage fast activity by 30–60 seconds [18]. 

Muscle atonia is partly the result of neurons in the dorsolateral PRF [8]. Through glutamatergic and/or GABAergic connections [19], these neurons project to the bulbar reticular formation (BRF) which inhibits lower motor neurons via GABA and glycine [20]. Muscle atonia is also the result of loss of serotonergic and noradrenergic tone as these neurotransmitter systems are silent in REM sleep [21–24].

Collectively the LDT, PPT, PRF, and MRF are known as the “REM-on” neurons. In contrast, there are populations of “REM-off” neurons mainly in the serotonergic midline raphe nuclei and noradrenergic locus coeruleus (LC) [25–28]. Of the raphe nuclei, the chief nucleus is the dorsal raphe (DRN) [29] but others (such as the linearis centralis [30], centralis superior [31], raphe magnus [32, 33], and raphe pallidus [34]) have been implicated. Furthermore, there are also aminergic populations in the anterior pontine tegmentum near the pontomesencephalic junction as well as other “stray” neurons throughout the brainstem with REM-off characteristics [8]. While firing rates of REM-on neurons are the highest in REM sleep, firing of the REM-off neurons is maximal in wakefulness [35]. 

The REM-on and REM-off neurons mutually antagonize each other. The model first proposed by McCarley and Hobson in 1975 [36] was characterized as a reciprocal interaction model based on Lotka-Volterra equations originally used to describe interactions between prey (i.e., REM-on) and predator (i.e., REM-off) populations. In this model, REM-on neurons initially grow exponentially by positively feeding back onto each other. At the same time, they activate REM-off neurons as a form of negative feedback. After being activated, REM-off neurons inhibit REM-on neurons and simultaneously exert negative feedback pressure on themselves.

With respect to anatomical and functional correlation of REM-on neurons in the model, there exists a positive feedback connection between LDT/PPT and PRF/MRF neurons [3]. Furthermore, a negative feedback connection has been found between LDT/PPT and LC neurons [37]. Regarding REM-off neurons, serotonin and noradrenaline (presumably from the DRN and LC, resp.) have been found to inhibit bursting LDT neurons [38]. There also exist negative feedback inhibitory recurrent collateral pathways for both the DRN and LC [8].

The reciprocal interaction model provides one method of explaining the 90-minute alternations between 30-minute REM sleep periods and NREM sleep periods over the course of a usual night. In order to account for the first shorter REM episode, which typically occurs 70–120 (on average 90) minutes after sleep onset, subsequent versions of the model have included a “limit cycle” modification [39]. 

Furthermore, the hormone orexin (also known as hypocretin), which is secreted by neurons in the lateral hypothalamus, additionally fine-tunes transitions into and out of REM sleep by diurnally gating REM sleep over the course of the entire sleep-wake cycle [53]. One potential mechanism is through strategic and selective excitation of REM-off neurons [8, 54–57]. Also manufactured in the lateral hypothalamus by neurons intermixed with orexin neurons [58–60], melanin-concentrating hormone is another recently discovered agent which may play a similar diurnal role through an inhibitory, rather than excitatory, mechanism [58, 61, 62].

## 3. Clinical Observations of Seizures in REM Sleep

Initial studies on the frequency of interictal and ictal events during REM sleep were largely anecdotal and consisted primarily of case reports. Studies were heterogeneous in terms of seizure/epilepsy classification (e.g., waking epilepsy, definitely symptomatic epilepsy), patient population (e.g., severity of epilepsy, use of antiepileptic drugs), use of the EEG (e.g., 10–20 system, montages, method of detecting abnormalities, inclusion of benign variants as abnormal features), use of the polysomnogram (PSG) (e.g., use of electromyography, definition of wakefulness and sleep stages), and outcome measures of both interictal and ictal events. Gradually, however, the methodology for recording and scoring became more standardized and this permitted comparison. 

In this review, the total number of events in wakefulness and each sleep stage was extracted for each study examined. Rates of interictal and ictal events in wakefulness and each sleep stage were also extracted. If rates were not explicitly provided, then they were calculated by dividing the number of events by the duration of wakefulness and/or each sleep stage when available. To facilitate comparison, each rate was then divided by the rate for REM sleep in order to determine an “indexed” rate. In averaging these indexed rates, each study was not treated equally. Rather, a “weighted mean” was produced by weighting each study based on the number of patients contained with each.

If individual sleep stages were not separated, then the same rate was used for each constituent stage (e.g., a combined N1/N2 rate was used individually as a rate for N1 and a rate for N2). With respect to numbers of events, this was divided equally among the constituent stages (e.g., a total of 33 seizures for N1/N2/N3 were counted as 11 for each stage). Formerly stage III and stage IV sleep were combined into stage N3 for analysis. Depending on the study, the definition of wakefulness may have included wake periods after sleep onset (WASO), nocturnal awakenings, morning awakenings, and/or samples of fully alert daytime wakefulness. As studies were divergent, statistical significance could not be calculated.

### 3.1. Focal Seizures

**Figure 1:**
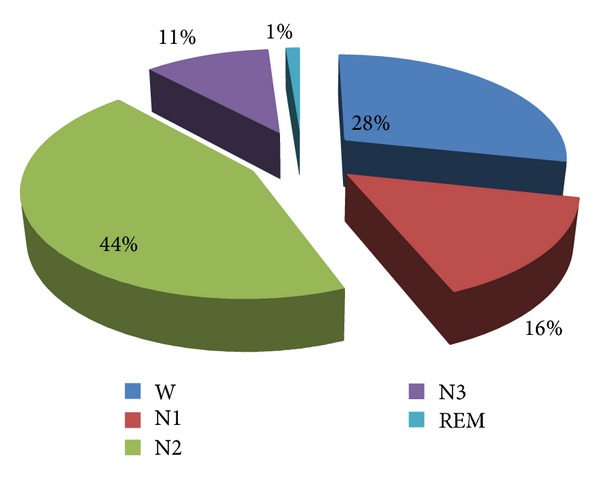
Raw sum of focal seizures.

A total of 542 patients with a collective 1990 seizures over 9 studies [40–43, 75–79] from 1987 to 2006 were included. Two studies [78, 79] were conducted using intracranial depth electrodes on patients classified with temporal or extratemporal epilepsy. The distribution of the 1990 seizures in wakefulness and specific sleep stages is shown in Figure 1.

The percentage of focal seizures during REM sleep over total recording time was extremely low (1%) over all these studies. However, because these studies did not provide specific durations, the length of recording may have led to artificial overinflation or underinflation of data. To address this issue, Table 1 provides a rate of focal seizure activity from four of these studies [40–43] where duration was provided.

Relative to REM sleep, the focal seizure rate was 7.83 times higher in wakefulness, 87 times higher in stage N1 sleep, 68 times higher in stage N2 sleep, and 51 times higher in stage N3 sleep. These data imply that focal seizures were most frequent in NREM sleep, intermediate in wakefulness, and lowest in REM sleep. However, the increased rate in wakefulness was highly variable with the weighted mean being powered by a single study [41]. In comparison, the study with the largest number of patients conducted by Herman et al. [43] yielded no seizures in either REM sleep or wakefulness.

### 3.2. Primary Generalized Seizures

A total of 256 patients with idiopathic generalized epilepsy were included among 7–9 studies [44–52] who ranged in age from 4 to 46. Specific subsets of idiopathic generalized epilepsy included juvenile myoclonic epilepsy, childhood absence epilepsy, and more generally “petit mal” and “grand mal” seizures in older studies. 

Table 2 demonstrates that, relative to REM sleep, the generalized discharge rate was 3.25 times higher in wakefulness, 3.1 times higher in stage N1 sleep, 3.13 times higher in stage N2 sleep, and 6.59 times higher in stage N3 sleep. In contrast to focal epilepsy, where data was available as shown in Table 3, no patients demonstrated maximal generalized discharges in REM sleep. 

### 3.3. Specific Epilepsy Syndromes

Benign epilepsy of childhood with rolandic spikes (BECRS) is the best studied focal syndrome in terms of discharge frequency in REM sleep. A total of 110 patients aged 3–16 were examined by Billiard et al. [63] and Dalla Bernardina et al. [64] in 2 separate studies. 

Table 4 demonstrates that, relative to REM sleep, discharges were about 4 times lower in wakefulness, about the same in stage N1 sleep, 1.1 times higher in stage N2 sleep, and 1.27 times higher in stage N3 sleep. In another study by Dalla Bernardina et al. in 1975 [80], comment was made that children with rolandic spikes but without seizures have a marked deactivation of discharges in REM sleep while those with seizures do not.

In addition to focal syndromes, a few case reports have also explored the impact of REM sleep on the epileptic encephalopathies. In 1981, Billiard et al. [70] presented 2 patients with Landau-Kleffner Syndrome aged 3 and 6 years who had higher interictal discharge rates in stage N1/2 (113% of REM) and N3 (127% of REM) sleep. However, Genton et al. [81] later published a case report of a 3.5-year-old girl with lateralized activation of right temporal spike-wave complexes in REM sleep.

In 1982, Tassinari [82] described 19 cases of electrical status epilepticus in slow wave sleep (ESES). He noted that, in REM sleep, all electrical status disappeared with only 3 instances of electrographic seizures resuming at the end of the REM sleep period. Similarly in 1981, Hrachovy et al. [83] described 32 patients aged 1–43 months with infantile spasms. He also noted that all patients demonstrated less or no hypsarrhythmia on the EEG during REM sleep.

### 3.4. Focal Interictal Discharges

A total of 214 patients were included among 7–10 different studies [47, 48, 65–73] who ranged in age from 2 to 61 years. Three studies [71–73] were conducted using intracranial depth electrodes on patients classified with temporal, frontal, occipital, parietal, or limbic seizures.

Table 5 demonstrates that, relative to REM sleep, the focal interictal discharge rate was 1.11 times higher in wakefulness, 1.75 times higher in stage N1 sleep, 1.69 times higher in stage N2 sleep, and 2.46 times higher in stage N3 sleep. These data imply that discharge rates are highest in NREM sleep (particularly stage N3) and comparable between wakefulness and REM sleep with the latter having a slightly lower firing rate.

Although REM sleep had the lowest rate of focal interictal discharges overall, this did not mean that each individual patient necessarily had lower rates of discharges in REM sleep. As Table 6 shows, where data was available, a weighted mean 10.9% of patients had maximal interictal discharge rates in REM sleep. This was second only to stage N3 sleep. In the context of the findings in Table 1, this implies that while these patients had maximal discharge rates in REM sleep compared to wakefulness or other sleep stages, the absolute rate remained low.

In intracranial depth recordings, Rossi et al. [71, 72] showed that, in REM sleep, there was a selective increase in the rate of interictal discharges over the epileptogenic zone (defined as the region which after resection resulted in seizure freedom) when compared to other sampled parts of the brain. While Wieser [84] commented on previous similar findings of increased REM sleep interictal discharge rates over the amygdala and supplementary motor area, he noted that these studies often considered benign variants as epileptiform [85, 86].

### 3.5. Selective Localization of Epilepsy in REM Sleep

Like seizure frequency in REM sleep, the impact of REM sleep on the distribution of interictal and ictal phenomena is controversial. The most powerful argument for clinically useful localization of an epileptogenic focus in REM sleep comes from a subset of tuberous sclerosis patients and a single temporal lobe epilepsy patient in studies by Ochi et al. [87] and Malow and Aldrich [88], respectively. In 6 of Ochi's patients, the semiology, neuroimaging, and other EEG were discordant in localizing the epileptogenic focus. Focal resection was undertaken in the hemisphere to which discharges were selectively lateralized in REM sleep. Four of six patients did well after surgery (Engel class I or II). In Malow's case report, 1 patient with bitemporal discharges selectively lateralized in REM sleep. After amygdalohippocampectomy in this hemisphere, the patient was rendered seizure-free for at least 3 years. For these patients, lateralization based on REM sleep alone was able to localize the epileptogenic zone in the midst of discordant data and predict seizure freedom.

Other studies have explored the localizing ability of REM sleep in relation to a “final” localization based on general concordance of all available data. 100% of unilateral temporal lobe patients with REM-lateralized interictal discharges or seizures were lateralized to the same hemisphere as the “final” localization. For NREM sleep, the concordance rate of interictal discharges and seizures was 100% and 94%, respectively. For wakefulness, it was a respective 88% and 94%. In patients where discharges were bitemporal, REM localization agreed with the final localization 100% of the time (compared to 81% in NREM and 100% in wakefulness). In an intracranial study by Lieb et al. [89], eight of 10 patients with REM-lateralized interictal discharges demonstrated statistically significant concordance with the final localization. Statistical significance for lower rates of concordance could not be established for wakefulness, “light sleep”, or “deep sleep”.

However, there remains controversy regarding the localizing value of interictal discharges. For example, in Lieb's study [89], two of 10 patients with REM-lateralized discharges were discordant with the final localization. Furthermore, Genton et al. [81] have described a case of Landau-Kleffner Syndrome in which spike rate dramatically increased and spread contralaterally during REM sleep. In an intracranial depth electrode study of 15 temporal lobe epilepsy patients, Montplaisir et al. [73] noted that spikes were often seen in areas outside the epileptogenic zone in the ipsilateral hemisphere as well as in homologous regions to the epileptogenic zone over the contralateral hemisphere.

## 4. How REM Sleep Could Affect Seizures

As previously discussed, the observed desynchronized EEG of REM sleep is the result of cholinergic MRF neurons depolarizing the thalamus which allows transmission of information to the cortex. Like REM sleep, the EEG of wakefulness is also desynchronized because cholinergic activity is likewise present. 

In contrast, cholinergic neurons are less active in NREM sleep with the least activity occurring in deep slow-wave sleep (i.e., stage N3) [10]. Without afferent mesencephalic cholinergic stimulation, the thalamus is not depolarized. An inactive thalamus does not allow transmission of information to the cortex. Without the inhomogeneous stimulation afforded by afferently transmitted information through the thalamus, cortical neurons are then able to intrinsically fire in a synchronized fashion. This is reflected by the maximally synchronized EEG of stage N3 sleep. 

To summarize, REM sleep and wakefulness represent states of maximal cortical synchrony, stages N1 and N2 sleep are states of intermediate synchrony, and stage N3 sleep represents a state of maximal cortical desynchrony.

### 4.1. Focal Interictal Discharges

When neurons exhibit asynchronous discharging behaviour at baseline, there is a reduced opportunity for spatial and temporal summation of any additional spontaneous depolarization [90, 91]. Such spontaneous depolarizations by populations of abnormally excitable neurons, in other words the “paroxysmal depolarizing shift”, have been hypothesized to be the mechanism behind focal epilepsy and interictal epileptiform discharges [92]. 

The reduced opportunity for spatial and temporal summation of such abnormal depolarizations may account for the results contained within Table 5. These data demonstrate that the highest rate of focal interictal discharges is in stage N3 sleep. This is in contrast to the lowest discharge rate which occurs comparably across REM sleep and wakefulness.

Another possible mechanism by which cortical desynchrony may account for this disparity in focal discharge rates is through the emergence of regional antiepileptic “microrhythms”. In contrast to the uniform global cortical synchrony of stage N3 sleep, there usually are distinct regional rhythms in more desynchronized states. For example, a posterior dominant alpha rhythm often exists in wakefulness. Even during the intermediately synchronized EEG of stage N2 sleep, there are regional sleep spindles located in the frontocentral regions bilaterally which, by definition, disappear with the onset of slow-wave sleep. Furthermore, a recent study has commented on “islands of hyperconnectivity” in REM sleep [93].

While the regional rhythms mentioned above are not known to be antiepileptic, another rhythm has been described, the hippocampal theta rhythm, which is also present in the desynchronized states of REM sleep and wakefulness [94]. In animal models, this rhythm has been shown to exert an antiepileptic effect [95]. 

However, the opposite may also be true and there could exist proepileptic regional rhythms in certain individuals. This may account for the interindividual variability in Table 6 which examined in which state of consciousness did a particular individual have the greatest rate of focal interictal discharging. Despite comparable overall rates of focal interictal discharges in REM sleep and wakefulness among patients from all studies included for analysis, a respective 5.9% and 10.9% of individuals achieved maximal discharge firing rates in wakefulness and REM sleep.

### 4.2. Focal Seizures and Generalized Epilepsy

Similar to focal interictal discharges, focal seizures are also hypothesized to arise from the “paroxysmal depolarizing shift” [96]. However, subsequent organization is required to sufficiently activate and recruit surrounding neurons in order to transform a focal interictal epileptiform discharge into an ictal event [96]. Recruitment of surrounding neurons leads to loss of surround inhibition, and seizure activity then spreads contiguously via local “short” cortical-cortical connections [96]. Secondary generalization may occur if there is spread to more distant areas via “long” association pathways such as the corpus callosum [96].

Like secondarily generalized seizures, primary generalized epilepsy also involves spread via long pathways but through a mechanism distinct from the paroxysmal depolarizing shift. Both primary generalized ictal and interictal phenomena have been hypothesized to be the result of abnormally synchronized and reverberating thalamocortical networks [97]. The distinction between ictal from interictal events rests mainly on discharge duration (i.e., greater than 10 seconds) and presence of clinical correlate [98].

Tables 1 and 2 demonstrate that rates of focal seizures and generalized discharges occur most often in NREM sleep when compared to either REM sleep or wakefulness. The same mechanisms proposed to account for a relatively higher rate of focal interictal discharges in NREM sleep would also apply to focal seizures and primary generalized epilepsy. 

Namely, the lower likelihood for spatial and temporal summation of aberrant spontaneous depolarizations in the cortically desynchronized states of REM sleep and wakefulness reduces the chance of spread along “short” pathways to surrounding neurons in focal epilepsy and “long” thalamocortical and association pathways in generalized epilepsy. Furthermore, should a desynchronized cortical milieu permit the emergence of regional antiepileptic microrhythms, this would present a further impediment to the spread of any aberrant depolarization.

However, unlike focal interictal discharges, the potential presence of proepileptic regional rhythms in certain individuals would not be expected to impact a primary generalized phenomenon. This is consistent with the results of Table 3 which demonstrate that among all patients in studies included for analysis, no individuals (0%) demonstrated a maximal discharge firing rate in REM sleep.

Returning to Tables 1 and 2, not only is it shown that rates of focal seizures and generalized discharges are lower in REM sleep and wakefulness, but rates are additionally lower in REM sleep compared to wakefulness. While both states of consciousness share a desynchronized EEG due to cholinergic activity, they differ greatly in terms of other neurotransmitter activity and in terms of connectivity.

Serotonergic neurons primarily located in the raphe nuclei, noradrenergic neurons in the locus coeruleus, and histaminergic neurons from the tuberomammillary nucleus demonstrate maximal firing rates in wakefulness and lowest firing rates during REM sleep [10]. These neurotransmitters are generally considered to produce arousal through widespread and usually excitatory effects on target neurons [10]. Such effects, present in wakefulness and absent in REM sleep, may account for recently discovered significant differences in connectivity between REM sleep and wakefulness. 

An fMRI study [99] of the default network demonstrated substantially reduced connectivity in REM sleep when compared to wakefulness. The greatest difference appeared to be disconnection of the dorsomedial prefrontal cortex. This was validated by another study [100] examining functional connectivity by multichannel EEG which disclosed disconnection of anterior from posterior cortical areas in REM sleep. Loss of the organizing influence from the frontal lobes may be reflected by the often illogical and nonsensical content of dreams in REM sleep [101]. 

Because a loss of connectivity precludes the presence of synchrony, strategic losses of brain connectivity in REM sleep compared to wakefulness might explain any extra antiepileptic effect of REM sleep. As previously discussed, the greater the degree of desynchronization, the less likely the spatial and temporal summation of any aberrant spontaneous depolarization which would allow “spread” along “short” or “long” pathways in the brain.

### 4.3. Specific Epilepsy Syndromes

From the aforementioned case reports on the epileptic encephalopathies, REM sleep has been noted to usually have an antiepileptic effect. As an encephalopathy is, by definition, a spread-out and diffuse process, the reduced potential for such spread in the desynchronized environment of REM sleep may explain this observed antiepileptic effect.

In contrast, Table 4 demonstrates that the rate of rolandic interictal discharges in BECRS is higher in REM sleep than wakefulness. Like Table 2 which demonstrates a higher maximal rate of focal interictal discharges in REM sleep for certain individuals, the finding in Table 4 can also be explained by the presence of a proepileptic regional rhythm which may promote interictal discharging in the rolandic region.

### 4.4. Selective Localization

From the reviewed studies involving multifocal (i.e., tuberous sclerosis [87]) and focal [89] (i.e., temporal lobe [74, 88]) epilepsy, interictal discharges during REM sleep usually have a greater predictive value in selectively localizing an epileptogenic focus. Like the postulated mechanisms behind a lower rate of focal seizures and generalized discharges in REM sleep, selective localization may also be explained by the reduced chance of an aberrant spontaneous depolarization spreading—be it from a lower probability of spatial and temporal summation in a desynchronized cortical environment or the emergence of regional antiepileptic microrhythms.

However, there are also clearly described instances of false localization in the literature [73, 81, 89]. Like the postulated mechanism behind a higher rate of interictal discharges in certain individuals and in certain syndromes such as BECRS, the presence of a proepileptic regional rhythm may skew the propagation patterns of focal interictal discharges so as to point to a false localization of the epileptogenic focus.

## 5. Conclusion

Sixty years after the discovery of REM sleep, a wealth of literature has commented on the effect of REM sleep on seizures. In our review, we have demonstrated that, compared to NREM sleep, REM sleep has a strong antiepileptic effect against focal interictal discharges, focal seizures, and generalized seizures. We also found that REM sleep has an additional antiepileptic effect compared to wakefulness against focal and generalized seizures. 

While cases of false localization have been described, REM sleep has been demonstrated to have promise in helping localize epileptogenic foci with possible translation into postsurgical seizure freedom. The potential selective localizing value of REM sleep may argue for the use of dedicated sleep recordings in the presurgical evaluation of epilepsy. 

Finally, we hypothesize that the impact of REM sleep on epilepsy is due to a maximally desynchronized EEG pattern which reduces the likelihood of spatial and temporal summation of aberrant depolarizations. Although at first glance similar to wakefulness, recent connectivity studies demonstrate a further strategic loss of connectivity in REM sleep which we hypothesize accounts for its unique antiepileptic influence on seizures. 

## Figures and Tables

**Table 1:** Relative focal seizure rates*.

Paper/sleep stage	W	N1	N2	N3	REM
Minecan et al. 2002 [40]	0.00	6.00	7.00	5.00	1.00
Crespel et al. 1998 [41]—FLE	133.42	14.59	14.59	14.59	1.00
Crespel et al. 1998 [41]—TLE	55.08	1.67	1.67	1.67	1.00
Terzano et al. 1991 [42]	0.00	5.52	2.16	3.77	1.00

Weighted mean	7.83	87.25	67.84	50.78	1.00

*Herman et al. 2001 [43] was included in weighted mean but could not be displayed as a relative rate because no seizures occurred in REM sleep.

**Table 2:** Relative generalized discharge rates.

Paper/sleep stage	W	N1	N2	N3	REM
Halász et al. 2002 [44]	8.14	14.53	10.47	3.49	1.00
Parrino et al. 2001 [45]		3.50	3.50	3.50	1.00
Horita et al. 1991 [46]	1.43	3.54	0.75	0.00	1.00
Autret et al. 1987 [47]/1997 [48]	1.37	1.50	1.50	2.25	1.00
Autret et al. 1987 [47]/1997 [48]—Pediatrics	2.30	3.66	3.66	4.91	1.00
Touchon 1982 [49]	5.05	3.26	0.10		1.00
Kellaway et al. 1980 [50]	1.68	5.63	5.63	5.63	1.00
Sato et al. 1973 [51]		3.32	16.04	43.45	1.00
Ross et al. 1966 [52]	4.94	3.12	3.94	11.06	1.00

Weighted mean	3.25	3.10	3.13	6.59	1.00

**Table 3:** % patients with maximal generalized discharges per state.

Paper/sleep stage	W (%)	N1 (%)	N2 (%)	N3 (%)	REM (%)
Horita et al. 1991 [46]	0.0	100.0	0.0	0.0	0.0
Sato et al. 1973 [51]		0.0	8.3	91.7	0.0
Ross et al. 1966 [52]	23.1	7.7	7.7	61.5	0.0

Weighted mean	22.8	14.8	5.9	56.4	0.0

**Table 4:** Relative rolandic discharge rates.

Paper/sleep stage	W	N1	N2	N3	REM
Billiard et al. 1990 [63]	0.54	1.19	1.19	1.19	1.00
Dalla Bernardina et al. 1982 [64]	0.22	0.97	1.08	1.28	1.00

Weighted mean	0.27	1.00	1.10	1.27	1.00

**Table 5:** Relative focal interictal discharge rates.

Paper/sleep stage	W	N1	N2	N3	REM
Clemens et al. 2005 [65]	1.52	2.50	1.85	2.67	1.00
Clemens et al. 2003 [66]	0.41	1.56	1.48	2.46	1.00
Ferillo et al. 2000 [67]		0.91	1.09	1.81	1.00
Malow et al. 1998 [68]		2.45	3.79	7.39	1.00
Malow et al. 1997 [69]		2.38	4.50	7.13	1.00
Billiard et al. 1981 [70]—Symptomatic	0.84	1.68	1.68	1.68	1.00
Billiard et al. 1981 [70]—Definite	0.70	1.43	1.43	1.43	1.00
Autret et al. 1987 [47]/1997 [48]	0.44	1.18	1.18	1.15	1.00
Rossi et al. 1984 [71, 72]*	1.17	1.33	1.33	1.18	1.00
Montplaisir et al. 1982 [73]*	1.21		2.43		1.00

Weighted mean	1.11	1.75	1.69	2.46	1.00

*Intracranial depth electrode study.

**Table 6:** % patients with maximal focal discharges per state.

Paper/sleep stage	W (%)	N1 (%)	N2 (%)	N3 (%)	REM (%)
Clemens et al. 2005 [65]	11.1	11.1	0.0	66.7	11.1
Ferrillo et al. 2000 [67]		19.4	11.1	61.1	8.3
Sammaritano et al. 1991 [74]	2.5	3.8	3.8	77.5	12.5

Weighted mean	5.9	8.7	5.1	69.5	10.9
